# Protein Kinase Expression of the AKT/mTOR Signaling Pathway in Peripheral Mononuclear Cells of Schizophrenia Patients: A Pilot Study

**DOI:** 10.3390/neurosci6040116

**Published:** 2025-11-17

**Authors:** Anastasiia S. Boiko, Ekaterina V. Mikhalitskaya, Elena G. Kornetova, Nikolay A. Bokhan, Svetlana A. Ivanova

**Affiliations:** 1 Mental Health Research Institute, Tomsk National Research Medical Center, Russian Academy of Sciences, Aleutskaya Str., 4, 634014 Tomsk, Russia; uzen63@mail.ru (E.V.M.); mental@tnimc.ru (E.G.K.); bohan.na@ssmu.ru (N.A.B.); 2 Psychiatry, Addictology and Psychotherapy Department, Siberian State Medical University, Moskovskiy Trakt, 2, 634050 Tomsk, Russia

**Keywords:** schizophrenia, cell signaling, protein kinases expression

## Abstract

A comprehensive study of the contribution of dysfunction AKT/mTOR signaling to the pathogenesis of schizophrenia is needed. The aim of the study is to determine the expression of the protein kinase AKT/mTOR signaling pathway in peripheral mononuclear cells (PMCs) of patients with schizophrenia. Determination of AKT1, mTOR, p70S6K, GSK3-α, and GSK3-β in mononuclears was performed on multiplex analyzers. Statistical data processing was carried out using SPSS. The critical significance level for the differences was 0.05. The study included 58 patients with schizophrenia (F20) and 60 healthy individuals. We found an increase in the expression of AKT1 and p706SK in PM׳s of patients (*p* = 0.006, *p* = 0.001). Analysis of kinase expression was carried out depending on clinical characteristics (type of course, leading symptoms and duration of the schizophrenia). Increased expression of GSK3-α and GSK3-β was detected in patients with a duration of disease more than 5 years (*p* = 0.019, *p* = 0.018). The AKT/mTOR signaling cascade may play a significant role in the pathogenesis of schizophrenia. We can assume that signaling pathways are involved in neurobiological processes and can be targets for new methods of pharmacotherapy, prognosis and diagnosis of mental disorders.

## 1. Introduction

Schizophrenia is a severe mental disorder characterized by positive and negative symptoms, neurocognitive impairment and behavioral disturbances [1,2]. Positive symptoms manifest themselves in the form of hallucinations and delusions; negative symptoms include flattening of emotions and avolition. Neurocognitive deficit is characterized by memory, attention, executive functions, and association disturbances. The described symptoms are characterized by either a continuous or episodic course, in which the latter, in contrast to the former, is manifested by exacerbations of schizophrenia episodes and then remissions. As the disorder develops, negative symptoms come to the forefront of the clinical picture in a significant proportion of patients. The disorder causes significant distress to both the patient and all persons, and places a significant burden on health care systems and society as a whole. The most active period of the disorder is the first 5 years after onset. During this period, the most pronounced biological disturbances and significant social losses for patients occur [3].

The study of the functioning of regulatory proteins of the main signaling pathways that are involved in the cascades of molecular processes of growth and differentiation of neurons, synaptic transmission, mechanisms of apoptosis, and cell survival in mental pathology is a direction in the field of clinical and fundamental medicine that is actively developing at the present time [4,5]. Evidence has been obtained demonstrating that dysregulation of intracellular signaling pathways plays an important role in the pathogenesis of schizophrenia [6,7,8].

One of the important areas of modern research is the study of the mechanisms of biological predisposition to these diseases. It is possible to identify biomarkers that have syndromic specificity by studying the relationship between pathogenetic mechanisms and clinical characteristics of diseases. Molecules that are markers of diseases, progression of disorders, vulnerability or risk factors at different stages of the disease are distinguished among these biomarkers [9].

The PI3K/AKT/mTOR signaling pathway in the central nervous system has become an important area of research over the past decade because these kinases take part in different processes, including developmental processes, regulation of neuro-, glial- and synaptogenesis, neuroprotection and control of short-term and long-term synaptic interactions that affect the mechanisms of memory, cognition, neural plasticity and behavior throughout life. The PI3K/AKT/mTOR cascade is represented by phosphoinositide 3-kinase (PI3K), AKT (also known as protein kinase B) and mTOR (mammalian target of rapamycin) kinases.

p70S6K (mTOR effector proteins) are responsible for biogenesis in ribosomes through phosphorylation of ribosomal protein S6. mTOR stimulation involves insulin-like growth factor and its receptors, the epidermal growth factor receptor family and their ligands, endothelial growth factor receptors and their ligands, estrogens and estrogen receptors, and the proteins RAS, ABL AMPK, which trigger reactions involving PI3K and Akt kinases and Raf/Ras proteins [10].

Stimulation of growth and proliferation of mTORC1 also requires a restructuring of cellular metabolism, providing the cell with the necessary amount of components for the construction of new cell membranes and nucleotides [11]. Uncontrolled activation of mTORC1 results in the inability of the cell to adequately respond to metabolic stress conditions due to the fact that the cells cannot reduce the activity of anabolism. As a result, cells with mTORC1 activation are hypersensitive to deficiency of glucose [12] and unsaturated fatty acids under hypoxic conditions [13].

mTOR kinase is a key regulator of protein synthesis, autophagy and metabolism. Dysregulation is observed in the development of many diseases, including neurodegenerative diseases [14]. Phosphoinositides generated by PI3K and phosphoinositide-dependent kinases work together to activate the protein kinase AKT, which in turn enhances mTOR-mediated protein translation. These protein kinases are inactive and are activated in response to tyrosine kinase or G protein-coupled receptors. Full activation of AKT1 requires phosphorylation at serine 473 by the mTORC2 molecular complex [15].

Protein kinases AKT1 and AKT2 regulate a number of cellular functions: cell growth and proliferation, apoptosis, angiogenesis and cellular metabolism. This is achieved due to the fact that AKT targets more than 100 proteins [16].

The GSK3 (glycogen synthase kinase 3) family consists of two isoenzymes (GSK3α and GSK3β). The activity of the serine/threonine kinase GSK3 is positively regulated by phosphorylation of tyrosine residues [17] and negatively regulated by the phosphorylation of serine [18].

The AKT protein plays an important role in the negative regulation of GSK-3β, which is active in the cell. Thus, BDNF can block GSK-3β through activation of AKT and promote neuronal polarization, neuronal axonal growth and branching. In addition to AKT, GSK-3β kinase is phosphorylated by protein kinases A and C, PrkG1, ILK and p70S6K.

There is substantial evidence demonstrating abnormalities in the PI3K/AKT/mTOR expression and activity in different animal schizophrenia models and postmortem probes of patients [19,20,21,22]. However, regarding the level of PI3K, AKT, mTOR, GSK3-α and GSK3-β in peripheral mononuclear cells (PMCs), the results are contradictory [23,24,25], possibly due to the heterogeneity of schizophrenia. There are no studies in the literature on the level of kinases in peripheral blood mononuclear cells in patients with schizophrenia depending on the clinical characteristics and duration of the disease.

The aim of the study is to investigate the expression of the main protein kinases of the AKT/mTOR signaling pathway in the peripheral mononuclear cells of patients with schizophrenia.

## 2. Materials and Methods

The study included 58 patients with schizophrenia. The control group consisted of 60 healthy individuals, matched by gender and age to the patients.

The study was approved by the bioethical committee of the Mental Health Research Institute of the Tomsk National Research Medical Center (Tomsk NRMC) (protocol No 165 from 18 September 2023) and was carried out in accordance with the ethical standards of the Declaration of Helsinki of the World Medical Association (1975, revised in Helsinki in 2024). All participants were informed about the purposes and possible complications of the study and signed written informed consent.

Criteria for exclusion of patients from the study: non-compliance with the age range; epilepsy and psychopathy in the form of decompensation; somatic and neurological diseases in the acute stage; inability of patients to comply with the protocol requirements.

Clinical symptoms were analyzed using the psychometric assessment scale for the severity of positive and negative syndromes PANSS (Positive and Negative Syndrome Scale) [26] in an adapted Russian version. The use of PANSS allows for a multidimensional and typological assessment of psychopathological symptoms in schizophrenia, taking into account the identification and recording of positive and negative symptoms.

Blood sampling for the study was carried out before the prescription of antipsychotic therapy upon admission to the hospital. Venous blood was collected from subjects in the morning on an empty stomach into heparin-containing tubes (BD Vacutainer). Mononuclear cells were isolated on a ficoll–urografin density gradient, followed by preparation of cell lysates using Lysis buffer for Multiplexing reagents (Merck Millipore, Darmstadt, Germany).

Measurement of protein kinases AKT1, mTOR, p70S6K, GSK3-α, and GSK3-β in cell lysates of PMC was carried out on multiplex analyzers MAGPIX and Luminex 200 (Core Facility “Medical Genomics”, Tomsk NMRC, Tomsk, Russia) using the Cell Signaling Multiplex Assay kit (11-Plex Total Akt/mTOR Magnetic Bead kit) (Merck Millipore, Darmstadt, Germany). The detection result is processed by a special program xPONENT (Luminex, Austin, TX, USA) with subsequent transfer of data to the calculation program MILLIPLEX Analyst 5.1 (Merck Millipore, Darmstadt, Germany). The signal is recorded as median fluorescence intensity (MFI) and is proportional to the number of molecules present in the sample. The obtained MFI results are normalized according to the “reference” protein GAPDH (Glyceraldehyde-3-phosphate dehydrogenase). Because GAPDH is consistently and stably expressed at high levels in all tissues, it has been well established as a protein of general cellular (non-specific) functions and is widely used as a control for protein normalization [27]. Normalization is performed by dividing the obtained signal for each analyte by the median fluorescence intensity obtained for GAPDH and multiplying by 100%.

The statistical power of the study sample was analyzed. With our parameters, the power is 0.95, which means that the number of subjects examined is sufficient to achieve 95% probability of correctly rejecting the null hypothesis at a significance level of 0.05 and an average effect size of Cohen’s w of 0.37.

Statistical processing using the SPSS program (version 23 for Windows) was used to analyze the obtained results. The data are tested for distribution type using the Kolmogorov–Smirnov test (with Lilliefors correction) and the Shapiro–Wilk test. The data obtained does not correspond to a normal distribution, so the results are presented as medians and quartiles, and nonparametric criteria are used for comparison. Comparison of data in the study groups was performed using the Mann–Whitney U test with Bonferroni correction for multiple comparisons. The statistically significant level of differences was accepted as less than 0.05.

## 3. Results

### 3.1. The Main Demographic and Clinical Characteristics of Participants

The main group of subjects included 58 patients with schizophrenia aged 18 to 65 years. Psychiatrists determined the diagnosis of F20 according to the International Classification of Diseases 10 (ICD-10). The distribution by gender in the patient and control groups was equal. The main clinical characteristics of the patients, including the duration of the disease, the type of schizophrenia course and the leading symptoms, are presented in Table 1.

### 3.2. Expression of Protein Kinases of the AKT/mTOR Signaling Pathway

Expression of protein kinases was assessed in PMCs of patients and controls. As noted in Section 2, GAPDH is used as a normalizing control, according to literature data and another study. However, we compared the MFI levels of this protein in schizophrenia patients and healthy individuals (Supplementary Table S1). Median fluorescence intensity levels did not differ significantly between the study groups (*p* = 0.448), confirming the validity of using GAPDH as a normalizing control.

**Figure 1:**
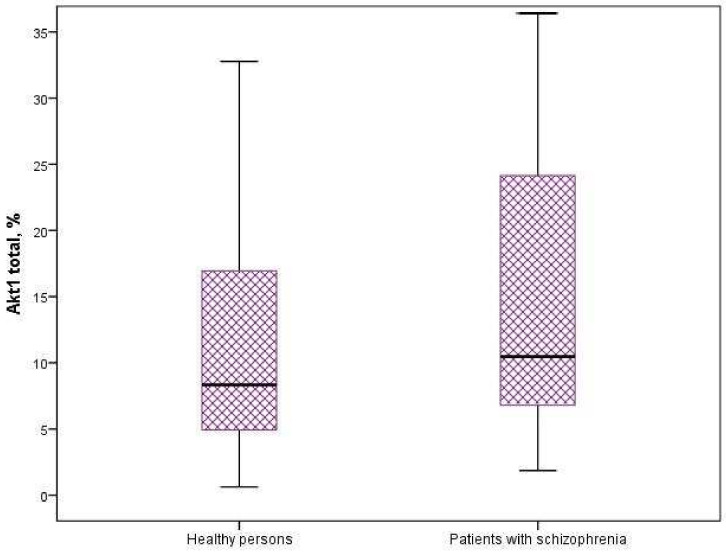
Expression of Akt1 in mononuclear cells of patients and healthy individuals (*p* = 0.006).

**Figure 2:**
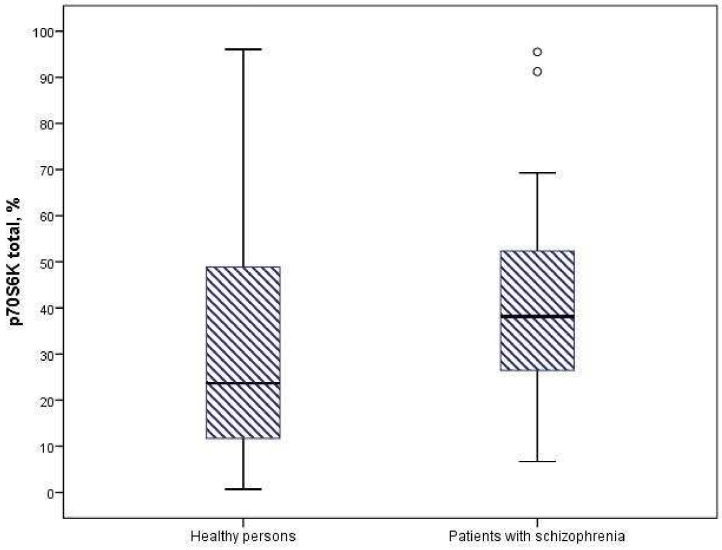
Expression of p70S6K in mononuclear cells of patients and healthy individuals (*p* = 0.001).

We found a significant increase in the expression of protein kinases AKT1 and p706SK in mononuclear cells of patients with schizophrenia compared to healthy individuals (*p* = 0.006 and *p* = 0.001) (Figure 1 and Figure 2). The intracellular content of mTOR, GSK3-α and GSK3-β kinases did not differ significantly in the compared groups (Table 2).

Further analysis of kinase expression in PMCs of patients with schizophrenia was carried out depending on the clinical characteristics: type of course (56.9% with continuous and 34.5% with episodic), leading symptoms (58.6% with negative and 41.4% positive) and duration of the disease (38% less than 5 years and 62% more than 5 years).

No significant differences were found in patients with schizophrenia with continuous and episodic course, as well as with negative and positive symptoms (Table 3 and Table 4).

The median duration of schizophrenia was 9 [5; 16] years. Increased expression of intracellular GSK3-α and GSK3-β was detected in schizophrenia patients with a disease duration of more than 5 years (*p* = 0.019 and *p* = 0.018) (Table 5).

Thus, our study demonstrates significant changes in the expression level of intracellular AKT1 and p706SK kinases in patients with schizophrenia. Moreover, the duration of the disease has a significant effect on the expression of GSK3-α and GSK3-β in schizophrenia patients.

## 4. Discussion

Any damage to brain tissue leads to the activation of neuroplasticity mechanisms, which is a response to pathological effects on neurons and compensatory and restorative mechanisms.

Changes in signaling activity in brain neurons are consistent with changes in peripheral blood mononuclears in patients, which can be used for effective diagnosis of diseases [28]. Our results regarding AKT are consistent with literature data on the association of this kinase with schizophrenia. There is evidence that the *AKT1* gene was initially identified as a potential susceptibility gene for schizophrenia [22]. Pregnenolone, an endogenous CNS neurosteroid, normalizes schizophrenia-like behavior via AKT signaling [29].

The increased expression of p70S6K kinase in peripheral blood mononuclear cells from patients with schizophrenia was found in comparison with controls and its content was ana-lyzed depending on the clinical characteristics. There are two studies in the literature that examine the plasma p70S6K level in vesicles but only in relation to insulin resistance in schizophrenia patients [30,31]. Fatemi S.H. et al. (2017) found significantly higher levels of p70S6K in the nuclear fraction of individuals with schizophrenia, but in the rough endoplasmic reticulum of individuals with schizophrenia, they identified significantly lower level of p70S6K [32]. This kinase is an integral part of the studied signaling pathway. We can speculate that PI3K/AKT/mTOR signaling may play a significant role in the pathogenesis of schizophrenia. Matsuda et al. (2019) came to similar conclusions in their review devoted to the study of the role of AKT/mTOR signaling pathway in the pathogenesis of mental disorders [28].

Altered GSK3 activity has also been implicated as a risk factor for schizophrenia [29]. There is substantial evidence demonstrating abnormalities in AKT expression and activity in different animal schizophrenia models and in the postmortem probes of patients [19,20,21]. However, the results are contradictory regarding the levels of mTOR, GSK3-α, and GSK3-β in peripheral blood cells. Both hypofunction of GSK3β in peripheral blood mononuclear cells of drug-naive patients with first-episode psy-chosis [23] and an increase in total and phosphorylated GSK-β levels in first-episode patients [24] have been de-tected. We did not find significant differences in mTOR, GSK3-α and GSK3-β expression between our chronic paranoid patient and control groups [25], and our results are consistent with those that found no differences in GSK3-α and GSK3-β levels in peripheral blood lymphocytes in chronic paranoid schizophrenia patients. The relationship between GSK3 kinases and the clinical polymorphism of mental disorders and the pharmacotherapy used has not been sufficiently studied [5]. Our results address this issue to some extent, as we found evidence of the association of GSK3-α and GSK3-β with important clinical characteristics such as duration of schizophrenia.

The analysis of literature data indicates a significant role of impaired functional activity of GSK-3β in the pathogenesis of schizophrenia [33] and affective disorders [34] and the possibility of using the kinase as a molecular target for the development of therapeutic strategies.

In our study, we determined the content of kinases in peripheral blood mononuclear cells. It is implied that the kinase expression levels in peripheral mononuclear cells reflect CNS signaling activity. Peripheral mononuclear cells are practical for biomarker studies and recent work has shown some correlation between peripheral markers and CNS molecular states. Gladkevich et al. (2004) suggest that blood lymphocytes, while offering a restricted view, can be a valuable tool for examining certain cellular processes, particularly gene activity [35]. Research has highlighted parallels between the way receptors are expressed and signaling pathways operate in cells of the nervous system, such as neurons and glial cells, and in lymphocytes. Neuropsychiatric disorders often involve changes in the metabolism and cellular processes within the central nervous system (CNS), alongside disruptions to crucial neurotransmitter and hormonal pathways. These disruptions are frequently accompanied by functional and metabolic abnormalities in blood lymphocytes. Emamian E.S. et al. (2004) investigated if lower AKT1 amounts led to a decrease in its target proteins in both blood lymphocytes and brain tissue from deceased individuals and their analysis revealed a similar pattern of change comparing blood samples with brain tissue [22]. Goossens et al. (2021) performed a comprehensive analysis of existing research and found that, despite challenges posed by study variations and limited data, certain properties of peripheral blood mononuclear cells appear to hold potential as clinical biomarkers for treatment response. These promising characteristics primarily involve inflammation processes and cell survival [36]. At the same time, we should be cautious in interpreting the results obtained in peripheral cells in relation to their translation on processes occurring in the brain due to the fact that evidence specific to intracellular kinase signaling is limited and inconsistent.

On the other hand, our results may provide novel insight into the peripheral dysfunction of the signaling pathways and highlight kinase expression as a prominent integrator of altered peripheral immune and metabolic states. The studied kinases are involved in the development of insulin resistance in schizophrenia [23]. Our previous studies, performed with the inclusion of the same patients, demonstrated activation of immune inflammation and a high incidence of metabolic syndrome [37,38]. One of the promising areas of future research is to study the relationship between kinase signaling pathways, immune inflammation and metabolic disorders in patients with schizophrenia.

At the same time, with our and similar studies of kinase levels in peripheral blood cells, there are many studies in the literature using RNAseq, microarrays and LCMS to measure these kinase genes in the postmortem brain. Analyzing gene expression differences identified alterations in the activity of genes associated with the PI3K-Akt signaling pathway and the complement system within the prefrontal cortex tissue samples taken after death from Japanese individuals diagnosed with schizophrenia [39]. A recent study by Devine e al (2025) reveals increased AKT1-3 mRNA levels in neurons of schizophrenia patients. This finding contradicts earlier research using postmortem tissue, which found either stable or reduced AKT protein levels, and this discrepancy implies a possible decoupling between mRNA and protein concentration [40]. Furthermore, investigations have highlighted the involvement of Akt signaling in the operations of genes linked to schizophrenia vulnerability, including *DISC-1, NRG-1* and *dysbindin-1* [41]. Consequently, abnormal AKT activity could foster an environment where genes associated with risk for schizophrenia can influence neuronal structure and function. Interestingly, the anterior cingulate cortex of individuals with schizophrenia exhibited the most significant AKT dysfunction, despite no clear link between alterations in kinase activity and corresponding changes in protein or phosphorylated protein amounts [42].

A cell’s unique identity is established through the varying amounts of proteins it produces. The correlation between the levels of mRNA and their corresponding proteins reveals how translation and protein breakdown, along with transcription and mRNA lifespan, collectively influence the control of gene expression [43]. New research indicates that the control of protein levels through transcription, RNA processing, translation and protein breakdown might be more significant than previously thought. In fact, variation mRNA levels alone accounts for less than half (40%) of the differences observed in protein quantities [44].

The functionality of kinases can be influenced by the presence of different gene variations. Our earlier research revealed for the first time a novel connection between specific gene polymorphisms related to neuroplasticity and protein kinases (including *MAPK, BDNF, GSK3β,* and *AKT1*) and the development of schizophrenia symptoms that predict a poor disease outcome [45]. McGuire J.L. et al. (2017) revealed that the *AKT1* genetic variation rs1130214, which has been linked to schizophrenia, affected the function of the AKT1 enzyme, although it did not alter the quantities of either the enzyme protein or its phosphorylated forms [42].

A number of limitations need to be mentioned. This is a pilot study, which is a small-scale preliminary study conducted to attract the attention to this topic; this means that, firstly, the groups of patients with different types of course, duration of the disease and leading clinical symptoms are small. Secondly, we did not take into account the influence of previous psychopharmacotherapy treatments of the patients in our study. Patients had previously taken second-generation antipsychotics, such as olanzapine, risperidone and quetiapine in therapeutic doses, but were not under our observation, so we cannot definitively state their adherence to treatment. At the same time, it is known that antipsychotics affect intracellular signaling. Antipsychotic medications influence various intracellular signaling pathways, including those involving cyclic adenosine monophosphate (cAMP), AKT/GSK-3 and MAPK. These interactions explain the diverse therapeutic effects and potential adverse reactions associated with different antipsychotic drugs [46]. As an illustration, haloperidol administration in mice results in a gradual rise in the phosphorylation level of AKT1 within brain cells [22]. Preclinical research suggests that both typical conventional and newer antipsychotic medications can suppress the function of GSK-β, albeit through indirect mechanisms [47]. Furthermore, these drug classes exhibit distinct impacts [48]. To gain a clearer understanding of these differences, long-term studies focusing on patients new to antipsychotic treatment are required; alternatively, investigations into kinase levels during single-drug therapy with either first- or second-generation antipsychotics would be beneficial. The third limitation is that we only examined the kinase levels in mononuclear cells; however, additional studies of mRNA are needed to study the mechanisms of signaling dysregulation, which will be the subject of further research.

## 5. Conclusions

The AKT/mTOR signaling cascade may play a significant role in the pathogenesis of schizophrenia. We can assume that these signaling pathways are involved in neurobiological processes and can be used for creating new methods of diagnosis and therapy of mental disorders.

## Figures and Tables

**Table 1:** Basic demographic and clinical characteristics of participants.

Parameter		Healthy Persons (n = 60)	Patients with Schizophrenia (n = 58)
Age, years		43 [30; 55]	35 [28; 42]
Gender, n (%)	Male	25 (41.7%)	32 (53.3%)
	Female	35 (58.3%)	28 (46.7%)
Duration of disorder, years		-	9 [5; 16]
Clinical course, n (%)	Episodic course	-	20 (34.5%)
	Continuous course	-	33 (56.9%)
	Not defined		5 (8.6%)
Leading symptomatology, n (%)	Negative	-	34 (58.6%)
	Positive	-	24 (41.4%)

Note: Data is presented as median [lower quartile; upper quartile] (Me [Q1; Q3]); not defined clinical course—observation period less than a year.

**Table 2:** Expression of protein kinases of the AKT/mTOR signaling pathway in mononuclear cells of patients with schizophrenia and healthy individuals (Me [Q1; Q3]).

Protein Kinases, %	Healthy Persons (n = 60)	Patients with Schizophrenia (n = 58)	*p*-Value
mTOR	3.99 [3.48; 5.23]	4.76 [3.28; 5.28]	0.56
GSK3-α	75.71 [56.15; 119.03]	86.21 [42.46; 209.99]	0.918
GSK3-β	39.55 [33.54; 71.7]	67.13 [19.65; 159.62]	0.732

Note: Data is presented as median [lower quartile; upper quartile] (Me [Q1; Q3]); comparisons between groups were performed using the Mann–Whitney U-test.

**Table 3:** Expression of protein kinases of the AKT/mTOR signaling pathway in mononuclear cells of patients with episodic and continuous course (Me [Q1; Q3]).

Protein Kinases, %	Continuous Course	Episodic Course	*p*-Value
AKT1	12.85 [6.17; 35.67]	11.67 [7.04; 21.16]	0.576
p70S6K	40.13 [26.41; 59.7]	37.59 [25.58; 44.74]	0.44
mTOR	4.81 [4.01; 6.82]	4.5 [2.09; 4.95]	0.2
GSK3-α	113.33 [54.27; 258.71]	83.14 [29.26; 150.3]	0.277
GSK3-β	80.2 [22.74; 191.01]	47.02 [16.13; 91]	0.423

Note: Data is presented as median [lower quartile; upper quartile] (Me [Q1; Q3]); comparisons between groups were performed using the Mann–Whitney U-test.

**Table 4:** Expression of protein kinases of the AKT/mTOR signaling pathway in mononuclear cells of patients with negative and positive symptomatology (Me [Q1; Q3]).

Protein Kinases, %	Negative Symptomatology	Positive Symptomatology	*p*-Value
AKT1	9.7 [6.16; 22.92]	10.94 [7.66; 38.53]	0.41
p70S6K	40.46 [27.63; 55.39]	37.53 [24.17; 50.41]	0.543
mTOR	4.77 [4.55; 5.33]	4.02 [2.27; 5.11]	0.237
GSK3-α	107.81 [64.43; 176.28]	62.34 [17.87; 226.23]	0.237
GSK3-β	74.66 [40.32; 99.82]	25.82 [9.65; 170.08]	0.203

Note: Data is presented as median [lower quartile; upper quartile] (Me [Q1; Q3]); comparisons between groups were performed using the Mann–Whitney U-test.

**Table 5:** Expression of protein kinases of the AKT/mTOR signaling pathway in mononuclear cells of patients depending on the duration of schizophrenia (Me [Q1; Q3]).

Protein Kinases, %	Less than 5 Year	More than 5 Years	*p*-Value
AKT1	8.37 [6.31; 21.16]	15.55 [6.62; 35.67]	0.345
p70S6K	39.56 [27.13; 57.32]	37.51 [24.73; 54.3]	0.588
mTOR	4.02 [3.05; 4.93]	4.77 [2.8; 5.31]	0.443
GSK3-α	46.21 [30.47; 96.69]	154.25 [85.44; 275.51]	0.019 *
GSK3-β	21.48 [12.32; 29.82]	93.27 [47.02; 201.48]	0.018 *

Note: Data is presented as median [lower quartile; upper quartile] (Me [Q1; Q3]); comparisons between groups were performed using the Mann–Whitney U-test. *—significant *p*-value < 0.05.

## Data Availability

The datasets are available on reasonable request to Svetlana A. Ivanova (ivanovaniipz@gmail.com), following approval of the Board of Directors of the MHRI in line with local guidelines and regulations.

## Supplementary Materials

The following supporting information can be downloaded at https://www.mdpi.com/article/10.3390/neurosci6040116/s1, Table S1: Level of MFI GAPDH in patients with schizophrenia and healthy individuals (Me [Q1; Q3]).

## Abbreviations

BDNF	brain-derived neurotrophic factor
TrkB	the receptor tyrosine kinase B
MAPK	mitogen-activated protein kinase
ERK	extracellular signal-regulated kinase
PLCγ	phosphoinositide phospholipase C
PI3K	phosphoinositide 3-kinase
AKT	also known as protein kinase B
mTOR	mammalian target of rapamycin
p70S6K	ribosomal protein S6 kinase
4E-BP1	initiation factor 4E binding protein 1
GSK3	glycogen synthase kinase 3
PANSS	Positive and Negative Syndrome Scale
MFI	median fluorescence intensity
GAPDH	glyceraldehyde-3-phosphate dehydrogenase
ICD-10	International Classification of Diseases 10
CNS	Central Nervous System
