# Supplementary material for: Protein Kinase Expression of the AKT/mTOR Signaling Pathway in Peripheral Mononuclear Cells of Schizophrenia Patients: A Pilot Study
Source: NeuroSci. 2025 Nov 17;6(4):116. doi: 10.3390/neurosci6040116 (PMC12641630; doi:10.3390/neurosci6040116)
Supplement: Supplementary file 1 [file neurosci-06-00116-s001.zip › neurosci-3759125-supplementary.pdf]

Supplementary Table S1. Level of MFI GAPDH in patients with schizophrenia and healthy individuals (Me [Q1; Q3]).

		Me [Q1; Q3]	p-value *
GAPDH, MFI	Healthy persons	1553 [1108; 2211.5]	0.448
	Patients with schizophrenia	1759 [1196; 2449]	

Note: MFI – median fluorescence intensity; * Comparisons between groups were performed using the Mann–Whitney U-test.
